# Serum irisin levels are lower in patients with breast cancer: association with disease diagnosis and tumor characteristics

**DOI:** 10.1186/s12885-015-1898-1

**Published:** 2015-11-11

**Authors:** Xeni Provatopoulou, Georgia P. Georgiou, Eleni Kalogera, Vasileios Kalles, Maira A. Matiatou, Ioannis Papapanagiotou, Alexandros Sagkriotis, George C. Zografos, Antonia Gounaris

**Affiliations:** 1 Research Center, Hellenic Anticancer Institute, Athens, Greece; 2 1st Department of Propaedeutic Surgery, Hippokratio Hospital, School of Medicine, University of Athens, Athens, Greece; 3 2nd Department of Surgery, Naval and Veterans Hospital of Athens, Athens, Greece

**Keywords:** Irisin, Serum levels, Breast cancer, Diagnostic indicator

## Abstract

**Background:**

Irisin is a recently discovered myokine, involved in the browning of white adipose tissue. To date, its function has been mainly associated with energy homeostasis and metabolism, and it has been proposed as a promising therapeutic target for obesity and metabolic diseases. This is the first study investigating the role of irisin in human breast cancer.

**Methods:**

Participants included one hundred and one (101) female patients with invasive ductal breast cancer and fifty one (51) healthy women. Serum levels of irisin, leptin, adiponectin and resistin were quantified in duplicates by ELISA. Serum levels of CEA, CA 15–3 and Her-2/neu were measured on an immunology analyzer. The association between irisin and breast cancer was examined by logistic regression analysis. The feasibility of serum irisin in discriminating breast cancer patients was assessed by ROC curve analysis. Potential correlations with demographic, anthropometric and clinical parameters, with markers of adiposity and with breast tumor characteristics were also investigated.

**Results:**

Serum levels of irisin were significantly lower in breast cancer patients compared to controls (2.47 ± 0.57 and 3.24 ± 0.66 μg/ml, respectively, *p* < 0.001). A significant independent association between irisin and breast cancer was observed by univariate and multivariate analysis (*p* < 0.001). It was estimated that a 1 unit increase in irisin levels leads to a reduction in the probability of breast cancer by almost 90 %. Irisin could effectively discriminate breast cancer patients at a cut-off point of 3.21 μg/ml, with 62.7 % sensitivity and 91.1 % specificity. A positive association with tumor stage and marginal associations with tumor size and lymph node metastasis were observed (*p* < 0.05, *p* < 0.01, *p* < 0.01, respectively).

**Conclusions:**

Our novel findings implicate irisin in breast cancer and suggest its potential application as a new diagnostic indicator of the presence of disease.

## Background

Irisin is a newly discovered myokine, secreted from muscle tissue as a cleavage product of fibronectin type III domain containing 5 (FNDC5), after shedding of the extracellular portion of the transmembrane protein into extracellular space [1]. It has a molecular weight of approximately 12 KDa and its amino acid sequence is highly conserved among most mammalian species, suggesting a highly conserved function [1]. Even though the predominant source of irisin is skeletal muscle, it was recently reported that adipose tissue also expresses and secretes irisin, suggesting that it may function not only as a myokine, but also as an adipokine [2]. Interestingly, irisin has a different pattern of secretion depending on the anatomical location of adipose tissue, with subcutaneous adipose tissue secreting more protein than visceral adipose tissue [2]. A recent comprehensive immunohistochemical study of irisin expression in human tissues indicated that the protein is locally produced in several central and peripheral tissues, potentially acting as a gatekeeper of metabolic energy regulation [3].

Irisin appears to exert a variety of functions, which are not yet fully elucidated. One of its main roles appears to be associated with the browning of white adipose tissue (beige cell formation), known to be involved in thermogenesis and energy expenditure [4, 5]. According to the proposed mechanism, skeletal muscle releases several myokines to the circulation during physical activity, including peroxisome proliferator-activated receptor Y coactivator 1α (PGC1α). The activation of PGC1α induces FNDC5 secretion, which is proteolytically cleaved to irisin. Irisin subsequently acts on both brown and white adipose tissue (BAT and WAT, respectively) [6]. Its primary effect on BAT is the activation of uncoupling protein 1 (UCP1) in mitochondria, resulting in the dissipation of energy in the form of heat also known as energy expenditure [7, 8]. The effect of irisin on WAT is the induction of BAT-like phenotypic changes. More specifically, it increases the expression of PGC1α and UCP1 as well as oxygen consumption, while it downregulates genes that are characteristic of WAT, process known as browning [1]. Altogether, these effects are associated with higher energy expenditure, which can further lead to the reduction of body weight and the improvement of metabolic parameters. As a result, irisin was originally proposed and investigated for its role as an exercise hormone and as a potential new agent for the treatment of obesity and metabolic diseases [9–18].

Obesity is a well-recognized risk factor for numerous diseases including metabolic, cardiovascular, and malignant diseases [19, 20]. Obese women are at an increased risk of breast cancer and typically present more aggressive disease, poorer outcomes and higher mortality rates [21–25]. A number of mechanisms have been proposed to mediate the link between obesity and breast cancer development, including adipose tissue-induced increased secretion of estrogens, insulin and insulin-like growth factors and altered production of adipokines [26, 27]. Adipokines have been recognized to participate in breast carcinogenesis providing a potential underlying molecular link between obesity and cancer development [28]. These factors can act on breast tissue in an endocrine, paracrine and autocrine manner exerting direct and indirect effects on breast cancer risk and progression [29–31]. Considering that alterations in adipokine secretion have been closely associated with breast cancer, it would be interesting to investigate the potential implication of irisin in disease development through its function as an adipokine.

The present study is the first attempt to explore the role of irisin in human breast cancer, by quantitatively determining serum levels of irisin in patients with invasive ductal breast cancer and healthy individuals. We aimed to examine the association between irisin and breast cancer and to evaluate the ability of serum irisin levels to discriminate between breast cancer patients and controls. We analyzed potential associations between irisin and various demographic, anthropometric and clinical parameters, as well as with established markers of obesity. Finally, potential correlations between irisin and breast tumor characteristics were assessed.

## Methods

### Participants

One hundred one (101) female patients with primary invasive ductal breast cancer were recruited from the 1st Department of Propaedeutic Surgery of Hippokratio Hospital of Athens upon disease diagnosis. In addition, fifty one (51) female healthy volunteers were recruited during their annual breast cancer screening, after exclusion of the presence of breast cancer or other suspicious breast lesions. Participants with other malignancies, impaired liver function, severe psychiatric conditions, cardiovascular diseases, metabolic diseases, diabetes, chronic kidney disease, diseases of the central nervous system, or under immunosuppressive agents were excluded from the study. Similarly, subjects under strenuous exercise within one month of the study were also excluded. Clinicopathological characteristics of patients and controls are presented in Table 1. The study protocol was approved by the Ethics Committee of the Hippokratio Hospital of Athens, Greece. All participants gave their written informed consent prior to entering the study.

**Table 1:** Baseline participants’ characteristics

	Controls	Cases	*p*-value
Demographic characteristics			
Ν	51	101	
Age (years)	55.7 ± 18.2	60.2 ± 13.7	0.131
Female gender	51 (100 %)	101 (100 %)	-
Menopausal status			0.183
Pre-menopausal	17 (34.0 %)	24 (23.8 %)	
Post-menopausal	33 (66.0 %)	77 (76.2 %)	
Anthropometric characteristics			
BMI (Kgr/m^2^)	25.7 ± 3.8	27.4 ± 5.1	0.021
BMI status			0.036
Normal weight	21 (42.0 %)	36 (36.4 %)	
Overweight	24 (48.0 %)	35 (35.4 %)	
Obese	5 (10.0 %)	28 (28.3 %)	
Chronic Diseases			
Dyslipidemia	23 (46.0 %)	13 (12.9 %)	<0.001
Cancer History			
Personal history of breast benign disease	3 (6.0 %)	4 (4.0 %)	0.685
Personal history of previous neoplasms	0 (0 %)	8 (7.9 %)	0.053
Family history of breast and/or gynecological cancer	7 (14.0 %)	38 (38.0 %)	0.002

### Sample analysis

Peripheral venous blood samples were collected from all patients preoperatively as well as from healthy controls. Serum samples were prepared by centrifugation according to standard protocols, aliquoted and stored at −80 °C until assayed. Irisin levels were quantitatively determined in duplicates using commercial enzyme-linked immunosorbent (ELISA) assays (AdipoGen International, Liestal, SW), according to the manufacturer’s instructions. Serum levels of leptin, adiponectin and resistin were also quantified by corresponding ELISA assays (BioVendor, Brno, CZ). Serum levels of cancer markers CEA, CA 15–3 and Her-2/neu were measured on an Advia Centaur Immunology Analyzer (Siemens, Tarrytown, US).

### Statistical analysis

Normality of distribution was evaluated through the Shapiro-Wilk test. Continuous variables are presented as mean ± standard deviation (SD) when they are normally distributed and as median (25th – 75th percentile) when they are skewed. Categorical variables are summarized as absolute (n) and relative frequencies (%).

Associations between categorical variables were tested by the use of contingency tables and the calculation of chi-square tests without the correction of continuity or Fisher’s exact test as appropriate. Associations between continuous variables and categorical variables with two categories were evaluated through Student’s t-test or Mann–Whitney when continuous variables were normally distributed or skewed, respectively. Associations between continuous variables and categorical variables with three or more categories were evaluated through one way ANOVA. However, due to multiple significance comparisons, we used the Bonferroni correction in order to account for the increase in Type I error.

Correlations between irisin levels and normally distributed continuous variables were evaluated by the Pearson’s correlation coefficient, while Spearman correlation coefficient was used to assess the correlation between irisin levels and skewed continues variables.

Logistic regression analysis was performed in order to determine whether the irisin levels are independently associated with the probability of women having breast cancer. Patients’ characteristics found to be significantly associated with the group were entered in the model as potential confounders. The results are presented as odds ratios (OR) and 95 % confidence intervals (95 % CI).

Cut-off point analysis was used to identify the optimal value of irisin levels that differentiates healthy women from women with breast cancer. The threshold was defined by the largest distance from the diagonal line of the receiver operating characteristic curve (ROC) (sensitivity x (1-specificity)). Using the cut-off points obtained from the analysis mentioned above, the sensitivity and specificity of the index for the aforementioned health outcomes were calculated. As sensitivity was defined the probability of a Ca patient having irisin level equal to or lower than a specific value, and as specificity was defined the probability of a healthy woman having irisin level higher than a specific value. A probability value of 5 % was considered statistically significant. All statistical calculations were performed on the SPSS version 21.0 software (SPSS Inc, Chicago, II, USA).

## Results

### Demographic characteristics

Table 1 presents baseline demographic characteristics of breast cancer patients and healthy controls. The two groups had no significant differences regarding age, gender and menopausal status. Significant differences were observed in anthropometric characteristics, as BMI was higher in cancer patients compared to controls (*p* = 0.021). A significant difference was observed regarding the presence of dyslipidemia, as a higher percentage of controls was dyslipidemic (*p* < 0.001). As far as cancer history is concerned, a significant difference was reported with a higher percentage of cancer patients having a family history of breast and/or gynecological cancer (*p* = 0.002), but not in personal history of benign breast diseases or previous neoplasms.

### Comparison of irisin levels between patients and controls

**Fig. 1:**
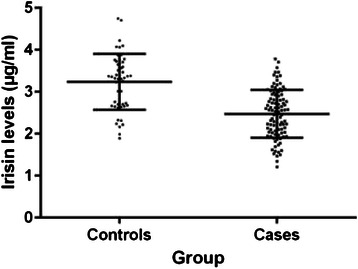
Scatter dot plots of serum irisin levels in patients with invasive breast cancer and controls. Mean values ± standard deviation are also denoted. Serum irisin levels were significantly lower in breast cancer patients compared to controls (2.47 ± 0.57 and 3.24 ± 0.66 μg/ml, respectively, *p* < 0.001)

Serum irisin levels were quantitatively determined in breast cancer patients and healthy controls. Cancer patients exhibited significantly lower serum levels of irisin compared to controls (2.47 ± 0.57 and 3.24 ± 0.66 μg/ml, respectively, *p* < 0.001) (Fig. 1). According to simple logistic regression analysis, there was a significant independent association between irisin levels and the presence of breast cancer (Table 2). It has been estimated that 1 unit increase in irisin levels results to almost 87 % reduction in the probability of women having breast cancer. The finding remained significant after controlling for potential confounders, and more specifically for BMI, dyslipidemia and family history of breast/gynecologic malignancy. After adjustment, it was estimated that 1 unit increase in irisin levels leads to almost 90 % reduction in the probability of women having breast cancer (Table 2).

**Table 2:** Association of serum irisin levels with the probability of women having breast cancer (Logistic regression analysis)

	Un-adjusted model		Adjusted model	
Patients’ characteristics	OR (95 % CI)	*p*-value	OR (95 % CI)	*p*-value
Irisin levels	0.131 (0.064–0.270)	<0.001	0.107 (0.045–0.255)	<0.001
BMI	-	-	1.117 (1.004–1.242)	0.041
Dyslipidemia	-	-	0.114 (0.039–0.330)	<0.001
Family history of breast/gynecological neoplasms	-	-	3.774 (1.114–12.784)	0.033

**Fig. 2:**
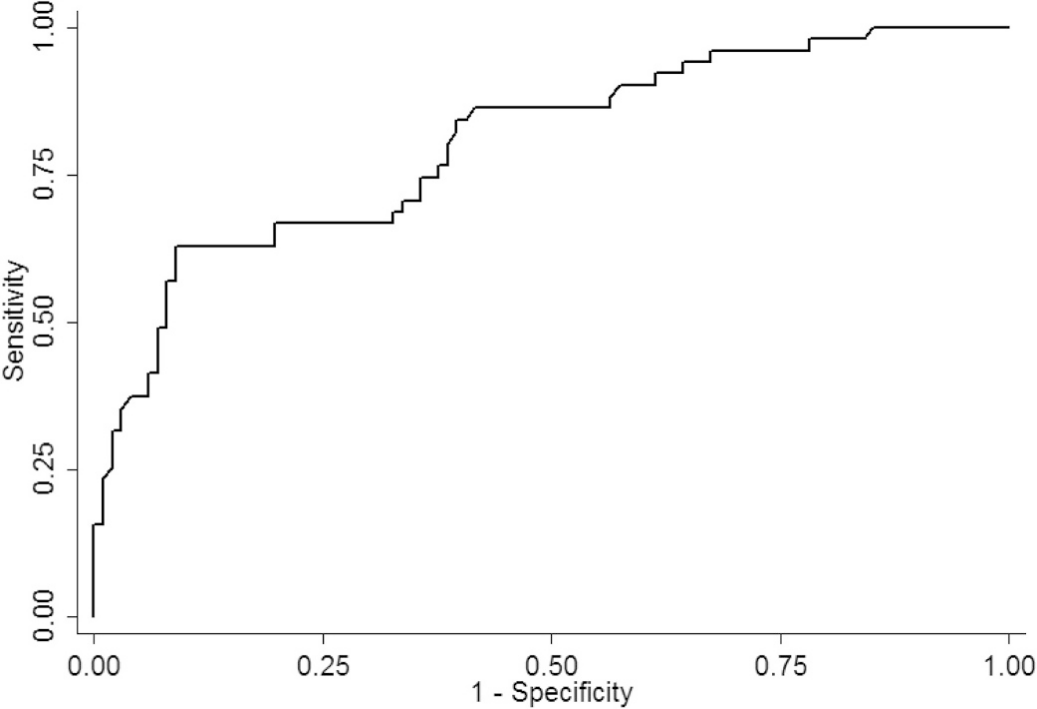
ROC curve analysis assessing the feasibility of serum irisin as a diagnostic indicator of breast cancer. Serum irisin can discriminate between breast cancer patients and healthy individuals at a cut-off point of 3.21 μg/ml, with 62.7 % sensitivity and 91.1 % specificity

Subsequently, ROC curve analysis was used to investigate the potential application of irisin for the discrimination between patients with breast cancer and healthy women. Our results indicate that irisin can effectively discriminate between patients and healthy individuals at an optimal value of 3.21 μg/ml. At this cut-off point, the sensitivity and specificity of detection is 62.7 and 91.1 %, respectively (Fig. 2).

### Αssociations between irisin levels and tumor/patient characteristics

Potential associations between serum irisin levels and tumor characteristics were investigated within the breast cancer group of patients (Table 3). Irisin levels were positively associated with tumor stage (*p* = 0.039), in particular stage I and stage III disease. Α marginal association with tumor size and lymph node metastasis was observed (*p* = 0.084 and *p* = 0.098, respectively). There was no significant association between irisin and tumor grade or the presence of concurrent DCIS. Irisin levels were not associated with ER, PR and c-erb-B2 status, or with Ki67 and p53 expression. No associations with serum levels of the established cancer markers CEA, CA15-3 and Her2/neu were observed (Table 4).

**Table 3:** Association between irisin levels and breast tumor characteristics

Characteristics	N (%)	Irisin levels (μg/ml)	*p*-value
Tumor size (cm)			0.084
≤2.0	43 (48.3 %)	2.34 ± 0.52	
2.1–5.0	31 (34.8 %)	2.58 ± 0.59	
>5.0	15 (16.9 %)	2.65 ± 0.57	
Grade			0.450
I	14 (14.3 %)	2.35 ± 0.59	
II	29 (29.6 %)	2.42 ± 0.59	
III	55 (56.1 %)	2.54 ± 0.56	
Stage			0.039
I	21 (30.4 %)	2.31 ± 0.54	
II	28 (40.6 %)	2.52 ± 0.58	
III	20 (29.0 %)	2.75 ± 0.50^a^	
Lymph nodes			0.098
Negative	40 (56.3 %)	2.44 ± 0.58	
Positive	31 (43.7 %)	2.66 ± 0.53	
Concurrent DCIS			0.161
No	34 (34.0 %)	2.37 ± 0.58	
Yes	66 (66.0 %)	2.54 ± 0.56	
ER status			0.966
Negative	24 (25.3 %)	2.46 ± 0.58	
Positive	71 (74.7 %)	2.46 ± 0.56	
PR status			0.904
Negative	30 (29.7 %)	2.47 ± 0.57	
Positive	65 (68.4 %)	2.46 ± 0.57	
c-erb-B2 status			0.226
Negative	64 (68.1 %)	2.41 ± 0.52	
Positive	30 (31.9 %)	2.56 ± 0.66	
Ki67 Value			0.243
<10 %	23 (24.5 %)	2.27 ± 0.54	
10–15 %	16 (17.0 %)	2.53 ± 0.67	
16–30 %	25 (26.6 %)	2.58 ± 0.54	
>30 %	30 (31.9 %)	2.49 ± 0.53	
P53 status			0.759
<10 %	52 (57.8 %)	2.45 ± 0.55	
>10 %	38 (42.2 %)	2.49 ± 0.57	

Data are presented as mean ± standard deviation

^a^compared to stage I after Bonferroni correction for multiple comparisons

**Table 4:** Associations between serum irisin levels, and serum levels of cancer biomarkers and of markers of adiposity

Biomarker	Median (25th–75th percentile)	rho	*p*-value
CEA (ng/ml)	1.36 (0.86–2.04)	0.082	0.455
CA 15–3 (U/ml)	15.90 (10.35–23.43)	−0.067	0.539
HER2/neu (ng/ml)	11.80 (10.05–14.90)	0.147	0.274
Adiponectin (μg/ml)^a^	12.30 ± 4.46	0.174	0.201
Resistin (ng/ml)	5.06 (4.35–5.94)	−0.203	0.134
Leptin (ng/ml)	18.99 (9.95–27.72)	0.399	0.003

^a^Data are presented as mean ± standard deviation

In breast cancer patients, irisin levels were not associated with BMI (*p* = 0.892). A positive association with leptin was observed, but not with adiponectin or resistin (Table 4). Finally, no associations between irisin and age, dyslipidemia or family history of breast and/or gynecological cancer were observed in the patients’ group (*p* = 0.098, *p* = 0.712 and *p* = 0.236, respectively).

## Discussion

Irisin is a muscle-derived factor, discovered and characterized three years ago [1]. Even though it is a relatively new molecule, a significant number of reports has already been published investigating its biology and function in several healthy and disease states. Originally, studies focused on the regulation of irisin by exercise with variable observations [1, 9–15]. At the same time, extensive research is being carried out on the role of irisin in metabolic diseases, while new findings regarding its implication in other chronic conditions gradually accumulate [32–42]. As far as the role of irisin in cancer is concerned, current data are scarce and only include in vitro reports [43, 44]. To our knowledge, the present study is the first aiming to investigate the potential role of the newly discovered irisin in human breast cancer. Our results demonstrate that serum irisin levels are significantly lower in breast cancer patients compared to healthy controls. Logistic regression analysis indicated that decreased irisin expression is closely associated with the presence of breast cancer. Most importantly, multivariate analysis showed that serum irisin is an independent predictor of breast cancer. It was estimated that 1 unit increase in irisin levels leads to almost 90 % reduction in the probability of women having breast cancer. According to ROC curve analysis, irisin can effectively discriminate between patients and healthy individuals with 62.7 % sensitivity and 91.1 % specificity at a cut-off point of 3.21 μg/ml, suggesting its potential value in breast cancer early detection.

Our observation of lower expression levels of irisin in breast cancer is supported by the findings of Gannon et al. evaluating the effect of various concentrations of irisin on the behavior of malignant and non-malignant breast epithelial cell lines [44]. The authors reported a significant suppressive effect of irisin on cell number, migration and viability in breast cancer cells, the induction of apoptotic cell death and the suppression of NFκB activity in malignant cell lines. Based on these data, they hypothesized that irisin may alter malignant characteristics similarly to other myokines and affect the development and aggressiveness of breast cancer [44]. It is thus reasonable to assume that reduced levels of irisin would favor breast cancer initiation, in agreement with our observations. The findings of Gannon et al. oppose previous data that reported lack of regulation of cell proliferation and malignant potential of other obesity-related cancer cell lines, including endometrial, colon, thyroid and esophageal, by irisin [43]. Nevertheless, these discrepancies have been attributed to different experimental techniques [44]. Since these are the only currently available data on the function of irisin in cancer, further studies are warranted to elucidate its molecular effects during disease development and to identify the implicated mechanisms.

Interestingly, a decreased expression of irisin has been associated with other chronic conditions, known to be associated with altered energy expenditure and metabolism. Most data arise from studies on patients with diabetes mellitus type 2 (DMT2), indicating a close relationship between the presence of disease and decreased circulating irisin levels [18, 32–34]. These observations are further supported by reports of down-regulation of the FNDC5 gene in these patients [18, 45]. Similarly, circulating irisin levels have been found significantly decreased in patients with chronic kidney disease (CKD) [35–37]. Since the development of DMT2 and CKD is often associated with insulin resistance, it has been suggested that irisin may play an important role in the pathology of insulin-resistance related disorders while it may provide a novel therapeutic target for the treatment of metabolic diseases [18, 32, 35]. Two recent reports provide evidence for the implication of irisin in the development of polycystic ovary syndrome (PCOS) and also suggest that it may be a marker of insulin resistance in these individuals [41, 42].

Adiponectin is another adipocyte-derived peptide hormone, able to stimulate the sensitivity of peripheral tissues to insulin, thus acting to enhance insulin sensitivity and protect against conditions of insulin-resistance [46]. Indeed, it has been documented that decreased levels of adiponectin are associated with increased insulin levels that accompany insulin resistance [47]. Low circulating levels of adiponectin have been observed in several diseases characterized by insulin resistance and hyperinsulinemia, including obesity, DMT2 and cancer [48–56]. Evidence from studies on breast cancer cell lines and animal models indicate that adiponectin can suppress cell proliferation, inhibit tumor growth, increase apoptosis and inhibit angiogenesis through multiple pathways [29–31]. Moreover, epidemiological data support that it exerts a protective role against breast cancer development, by displaying anti-proliferating, pro-apoptotic, anti-estrogen and anti-inflammatory properties [31]. More specifically, several studies on breast cancer patients have observed reduced adiponectin expression, particularly in postmenopausal women, suggesting a close association with breast cancer risk and providing a potential mechanistic link between obesity and breast cancer [53–55, 57–60]. Among the many mechanisms proposed to mediate adiponectin function in breast cancer, the mitogenic effect of hyperinsulinemia appears to be of particular importance [57]. According to our results, serum levels of both irisin and adiponectin were lower in breast cancer patients compared to controls (data not presented) but there was no significant association between them. Current knowledge on the functions of irisin and the mechanisms involved in exerting its actions is exceptionally limited and only allows for hypotheses to be made. Nevertheless, the decreased expression levels of both molecules in our cohort of patients support that irisin may play a role similar to that of adiponectin. Moreover, irisin’s downregulation in conditions characterized by insulin resistance suggest that this mechanism may be a major determinant of its function. Whether the effect of irisin on breast cancer can be attributed to obesity-related hyperinsulinemia and insulin resistance extends beyond the scope of this work, but is a particularly interesting field of research that is worthy of thorough investigation in future studies.

Interestingly, we found that irisin levels were higher in stage III disease compared to stage I, resulting in a positive association with tumor stage. Based on our observation of reduced levels of irisin in breast cancer compared to controls, a further downregulation with the extent or progression of the disease would possibly be expected. This, however, may be a rather simplified hypothesis. Cancer is an exceptionally complex disease, accompanied by major dysregulation of a vast number of interacting molecular mechanisms and signaling pathways. At present, it is still impossible to know how irisin may behave and interact either in physiological conditions or in disease states. One assumption for our observation would be that, after the establishment of breast cancer, the secretion and metabolism of irisin may be affected by other, yet unidentified, cancer-related pathways and factors, resulting in alterations in its expression levels. Otherwise, irisin might exert a different function trying to counteract disease progression that is reflected in its expression levels. Nevertheless, it should be noted that even at stage III disease, irisin levels remained significantly lower than in healthy controls. Since this the first approach, our findings arise from a relatively restricted patient group and their verification in larger cohorts of patients is mandatory.

According to our findings, irisin levels were not associated with serum levels of the established cancer markers CEA, CA15-3 and Her2/neu. Despite their widespread use in routine clinical practice, it is well-recognized that the traditional tumor markers are only suitable for detection of recurrent or metastatic breast cancer as well as for management and surveillance of patients with advanced disease [61–63]. Their lack of specificity and sensitivity for in situ and primary breast cancer bears serious limitations and renders them rather inappropriate biomarkers for screening and diagnosis of low-volume disease [61, 63]. Thus, our observation of no association between irisin and cancer markers is rather expected and may further support the potential diagnostic value of irisin.

Aiming to explore a potential link between irisin and obesity in human breast malignancy, we investigated potential correlations of irisin levels with body mass index (BMI) and with levels of the adipokines leptin, adiponectin and resistin in breast cancer patients. Our results indicate that irisin was positively associated with leptin, but not with adiponectin, resistin or BMI. Previously, several studies have investigated irisin expression in relation to obesity. BMI is the most traditional and widely-used measure of obesity. It has not yet been clarified if irisin levels are associated with BMI, as some studies have found positive correlations while others negative or no correlation [9, 10, 16–18]. It was recently proposed that irisin is positively correlated to BMI in healthy individuals but negatively correlated in subjects with metabolic diseases, suggesting a different function according to the metabolic state [64, 65].

Leptin is the most prominent and best studied adipokine, and has been suggested to be implicated in the development of hormone-dependent malignancies including breast cancer [66]. Many studies have investigated leptin as a risk factor for breast cancer mainly in postmenopausal women, with conflicting and opposing findings [67–73]. At the same time, few reports have explored the role of leptin during disease progression and/or in relation to its clinical behavior. Although data are diverse, studies have provided evidence that increased expression of leptin in patients with breast cancer is associated with large tumor size, advanced tumor stage, high tumor grade and lymph node metastasis [59, 60, 67, 74–76]. These observations have led to hypotheses that leptin is not only involved in breast cancer initiation but also in disease progression, and that it may be associated with more aggressive phenotypes [59, 60, 67, 74, 75]. In addition, leptin has been associated with distant metastasis and short survival, suggesting that it may have a negative prognostic value [76, 77]. Considering the potential relationship of leptin expression with the characteristics and biological behavior of breast tumors, we consider the association between irisin and leptin levels in our patients is a reflection of the positive correlations of irisin with tumor stage and marginally with tumor size and lymph node involvement. The association of irisin levels with the presence of disease and tumor characteristics but not with BMI suggests that this factor is more likely to be related to systemic disease and tumor extent rather than to adiposity, as previously reported for leptin [76].

Altogether, our findings provide preliminary data for the implication of irisin in breast cancer development and suggest that it may serve as a potential novel biomarker of the presence of disease. Cancer serum markers may have a number of clinical applications including early diagnosis, differential diagnosis, prognosis, prediction of response and resistance to therapy, patient monitoring and surveillance. In breast cancer, established tumor markers are characterized by limited specificity and sensitivity precluding their use for screening and early diagnosis [60, 63]. Mammography, in combination with clinical examination and breast self-examination, remains the primary modality for breast cancer screening and early detection of the disease, and has significant contribution to the decrease in mortality rates [78]. Nevertheless, mammography has important limitations particularly regarding the detection of suspicious lesions in women with very dense breasts and the early diagnosis of interval tumors [78]. As a result, improvements in the detection and early diagnosis of breast cancer are required and are expected to have a major impact on morbidity and mortality from the disease. In that aspect, intensive research is being carried out to exploit the use of novel serum biomarkers in screening, early diagnosis and differential diagnosis of breast cancer [79]. The identification of irisin, as well as other novel proteins, as candidate biomarkers that could assist in the early detection of the disease is an exciting perspective, and remains to be further investigated and verified by future prospective studies.

## Conclusions

Significant evidence implicating irisin in various diseases, both metabolic and other chronic conditions, are gradually accumulating. Nevertheless, there is still lack of knowledge on its exact function and mode of action either in healthy or in disease states. To our knowledge, this is the first study attempting to investigate the role and clinical relevance of irisin in human breast cancer. Our findings provide significant preliminary evidence that serum irisin may serve as a novel indicator for breast cancer detection and early diagnosis. Forthcoming studies aimed to clarify these aspects are awaited with anticipation and are expected to add significant benefit for these patients.

## Abbreviations

ANOVA: Analysis of variance
BAT: Brown adipose tissue
BMI: Body mass index
CA 15–3: Cancer antigen 15–3
CEA: Carcinoembryonic antigen
CI: Confidence interval
CKD: Chronic kidney disease
DMT2: Diabetes mellitus type 2
ELISA: Enzyme-linked immunosorbent assay
ER: Estrogen receptor
FNDC5: Fibronectin type III domain containing 5
HER-2/neu: Human epidermal growth factor receptor 2
ml: Milligram
μg: Microgram
NFκB: Nuclear factor-kappaB
OR: Odds ratio
PCOS: Polycystic ovary syndrome
PGC1α: Peroxisome proliferator-activated receptor Y coactivator 1α
PR: Progesterone receptor
ROC: Receiver-operating characteristic
SD: Standard deviation
UCP1: Uncoupling protein 1
WAT: White adipose tissue
